# Supplementary material for: Molecular Epidemiology of Mitochondrial Cardiomyopathy: A Search Among Mitochondrial and Nuclear Genes
Source: Int J Mol Sci. 2021 May 27;22(11):5742. doi: 10.3390/ijms22115742 (PMC8197938; doi:10.3390/ijms22115742)
Supplement: Supplementary file 1 [file ijms-22-05742-s001.zip › ijms-1167202-supplementary.pdf]

Supplementary data: Mitochondrial disease

Because mitochondrial OXPHOS is ubiquitous and crucial for cellular function, Mitochondrial disease (MDs), commonly, show multi-organ involvement, resulting in complex multisystem diseases. Furthermore, deficit of OXPHOS system may result from a single gene mutation, which can be expressed with different phenotype in families or members of the same family, but also different mutations can produce a similar clinical manifestation, thus complicating the establishment of diagnosis. MDs arise in child and adulthood and are due to both mitochondrial DNA (mtDNA) and nuclear genome (nDNA) alterations (prevalence: 9.6/100,000 and 2.9/100,000, respectively). In MDs adult onset, mtDNA mutations are more frequent, although childhood severe autosomal recessive disorders, due to mutations in nuclear genes, such as DNA polymerase gamma gene (POLG), involved in mtDNA replication, and Twinkle helicase gene (PEO1), may occur later in life. Mutations in mtDNA account for the 80% of MDs in adults and for 20-25% in childhood, where the majority of mitochondrial disorders affect nuclear variations.

- Ng, Y. S.; Turnbull, D. M., Mitochondrial disease: genetics and management. Journal of neurology 2016, 263, (1), 179-91.
- Naing, A.; Kenchaiah, M.; Krishnan, B.; Mir, F.; Charnley, A.; Egan, C.; Bano, G., Maternally inherited diabetes and deafness (MIDD): diagnosis and management. Journal of diabetes and its complications 2014, 28, (4), 542-6.
- Jameson, E.; Morris, A. A. M., Mitochondrial disease – a review. Paediatrics and Child Health 2011, 21, (2), 80-83.
- Schaefer, A. M.; Walker, M.; Turnbull, D. M.; Taylor, R. W., Endocrine disorders in mitochondrial disease. Molecular and cellular endocrinology 2013, 379, (1-2), 2-11.
- El-Hattab, A. W.; Scaglia, F., Mitochondrial Cardiomyopathies. Front Cardiovasc Med 2016, 3, 25.
- Mazzaccara, C.; Iafusco, D.; Liguori, R.; Ferrigno, M.; Galderisi, A.; Vitale, D.; Simonelli, F.; Landolfo, P.; Prisco, F.; Masullo, M.; Sacchetti, L., Mitochondrial diabetes in children: seek and you will find it. Plos One 2012, 7, (4), e34956.
- Murphy, R.; Turnbull, D. M.; Walker, M.; Hattersley, A. T., Clinical features, diagnosis and management of maternally inherited diabetes and deafness (MIDD) associated with the 3243A>G mitochondrial point mutation. Diabetic medicine: a journal of the British Diabetic Association 2008, 25, (4), 383-99.

Supplementary data: Mitochondrial genome (mtDNA)

The mtDNA spans 37 genes, coding for two ribosomal RNAs (rRNAs), 22 transfer RNAs (tRNAs) and 13 proteins of the multi-enzyme complexes, involved in the OXPHOS process, namely the NADH-ubiquinone oxidoreductase chain 1 (MTND1), chain 2 (MTND2), chain 3 (MTND3), chain 4 (MTND4), chain 4L (MTND4L), chain 5 (MTND5) and chain 6 (MTND6) of complex I; the Cytochrome b (MTCYTB) of complex III; the Cytochrome C oxidase 1 (MTCOX1), 2 (MTCOX2) and 3 (MTCOX3) of complex IV: and the ATP synthase subunit 6 (MTATP6) and subunit 8 (MTATP8) of complex V. Complex II is entirely encoded by the nuclear genome. The two filaments constituting the circular mtDNA molecule are the H chain (heavy), which encloses 28 genes (2 coding for rRNA, 14 for tRNA and 12 for proteins) and the L chain (light), including 9 genes (8 tRNA-coding genes and 1 coding gene for a polypeptide).

The mitochondrial genome of mammals has an extremely compact organization, without introns. Some protein-coding genes have overlapping sequences (ATP8 and ATP6 have 46 common bases,

ND4L and ND4, 7 common bases). The intergenic regions are limited to two non-coding (NC) areas: the first, called D-LOOP, or control region, is a short triple-helix trait of 1121 bps, located between the MT-TF and MT-TP genes, encoding the tRNA phenylalanine and the tRNA proline, respectively; it encompasses the main regulating sites of mtDNA replication and transcription. The second NC region is located between the MT-TN and MT-TC genes, encoding the tRNA asparagine and tRNA cysteine, respectively, and contains the origin of the L filament replication. These regions show typical nucleotide variants as useful markers determining mitochondrial haplogroups. A haplogroup is a combination of shared nucleotide variants (haplotypes) closely linked and together inherited. The haplogroup determination allows to have information regarding the identity attribution and degrees of kinship and to trace the origins of humankind. Furthermore, the identification of particular haplogroups also seems to have a diagnostic value, recognizing an increased/reduced risk of developing certain disease such as idiopathic dilated cardiomyopathy [40-43].

The mtDNA differs from nDNA in mode of inheritance, mutation rate and polyploidy; the mtDNA transmission does not follow Mendel's laws, but it is maternally inherited.

The absence of histones and of an efficient DNA repair system, as well as the continuous exposure to reactive oxygen species (ROS), produced by the mitochondrial respiratory chain (RC), are the major factors responsible for the high mtDNA rate mutations (10-20 times higher than nDNA). However, recent studies suggest that mtDNA is organized in protein complexes called nucleoids, such as the high mobility group (HMG) box family and the mitochondrial transcription factor A (TFAM), which appear to have a protective role on the mitochondrial genome. Furthermore, human cells contain, on average, several hundred mitochondria, depending on the energy needs for the tissue, each containing multiple copies (between two and ten) of a 16.6 kb circular double-stranded DNA. Thus, each cell contains thousands of mtDNA molecules. This feature of mtDNA gives rise to coexistence of mutant and wild type mtDNA molecules in one cell, defined as "heteroplasmy" The heteroplasmy level is crucial in determining cellular dysfunction, depending on the mutation and cell type. The threshold value, at which the pathology may occur, is typically, >50% (on average between 60% and 80%). Although some clinical disease manifestations are reported with lower heteroplasmy level (also 10%), very high levels are, generally, required for mutations interesting mt tRNA, on the contrary, single or large-scale mtDNA deletions produce RC defects when ~60% of mtDNA is deleted.

During oogenesis, to each egg cell is randomly assigned a small number of mtDNA molecules that will then be replicated to obtain the final number of mtDNA copies in the egg cell. This random assignment process leads the children of a heteroplasmic woman to have different proportions of mutated mtDNA in tissues. This phenomenon explains the phenotype heterogeneity in different individuals of the same family, having low or high proportion of mutated mtDNA molecules, so that the tissue can be considered wild type or affected, respectively.

In the presence of a heteroplasmic mutation, pathological dysfunction will occur when the ratio between mutated and normal mtDNA exceeds a threshold value, which is lower the higher is the energy demand of the affected tissue. Moreover, same mitochondrial disorders (i.e. LHON) show typically a homoplasmy condition instead of variable heteroplasmy.

- Lee, S. R.; Kim, N.; Noh, Y. H.; Xu, Z.; Ko, K. S.; Rhee, B. D.; Han, J., Mitochondrial DNA, mitochondrial dysfunction, and cardiac manifestations. Front Biosci (Landmark Ed) 2017, 22, 1177-1194.
- Limongelli, G.; Masarone, D.; D'Alessandro, R.; Elliott, P. M., Mitochondrial diseases and the heart: an overview of molecular basis, diagnosis, treatment and clinical course. Future cardiology 2012, 8, (1), 71-88.

- Limongelli, G.; Masarone, D.; Pacileo, G., Mitochondrial disease and the heart. Heart 2017, 103, (5), 390-398.
- Chinnery, P. F., Mitochondrial disease in adults: what's old and what's new? EMBO molecular medicine 2015, 7, (12), 1503-12.
- Dames, S.; Chou, L. S.; Xiao, Y.; Wayman, T.; Stocks, J.; Singleton, M.; Eilbeck, K.; Mao, R., The development of next-generation sequencing assays for the mitochondrial genome and 108 nuclear genes associated with mitochondrial disorders. The Journal of molecular diagnostics : JMD 2013, 15, (4), 526-34.
- Vesterkaer, S. M.; Baandrup, U., Mitochondrial cardiomyopathy. Virchows Arch 2013, 463, (2), 193-194.
- Imai-Okazaki, A.; Kishita, Y.; Kohda, M.; Mizuno, Y.; Fushimi, T.; Matsunaga, A.; Yatsuka, Y.; Hirata, T.; Harashima, H.; Takeda, A.; Nakaya, A.; Sakata, Y.; Kogaki, S.; Ohtake, A.; Murayama, K.; Okazaki, Y., Cardiomyopathy in children with mitochondrial disease: Prognosis and genetic background. International journal of cardiology 2019, 279, 115-121.
- Choi, Y.; Lee, J. H.; Cui, M. N.; Lee, Y. S.; Jung, M. H.; Yi, J. E.; Jung, H. O.; Youn, H. J., Hypertrophic Cardiomyopathy Attributable to Mitochondrial DNA Mutation Diagnosed by Pathology and Gene Sequencing. Circulation 2016, 133, (13), 1297-1299.
- Govindaraj, P.; Khan, N. A.; Rani, B.; Rani, D. S.; Selvaraj, P.; Jyothi, V.; Bahl, A.; Narasimhan, C.; Rakshak, D.; Premkumar, K.; Khullar, M.; Thangaraj, K., Mitochondrial DNA variations associated with hypertrophic cardiomyopathy. Mitochondrion 2014, 16, 65-72.
- Brecht, M.; Richardson, M.; Taranath, A.; Grist, S.; Thorburn, D.; Bratkovic, D., Leigh Syndrome Caused by the MT-ND5 m.13513G>A Mutation: A Case Presenting with WPW-Like Conduction Defect, Cardiomyopathy, Hypertension and Hyponatraemia. JIMD reports 2015, 19, 95-100.
- Fassone, E.; Rahman, S., Complex I deficiency: clinical features, biochemistry and molecular genetics. Journal of medical genetics 2012, 49, (9), 578-90.
- Alston, C. L.; Ceccatelli Berti, C.; Blakely, E. L.; Olahova, M.; He, L.; McMahon, C. J.; Olpin, S. E.; Hargreaves, I. P.; Nolli, C.; McFarland, R.; Goffrini, P.; O'Sullivan, M. J.; Taylor, R. W., A recessive homozygous p.Asp92Gly SDHD mutation causes prenatal cardiomyopathy and a severe mitochondrial complex II deficiency. Hum Genet 2015, 134, (8), 869-79.

Supplementary data: online database

National Library of Medicine (*https://www.nlm.nih.gov/*), Human Genome Mutation Database HGMD Professional (*https://portal.biobase-international.com/hgmd/pro/search_gene.php*), ClinVar (*https://www.ncbi.nlm.nih.gov/clinvar/*), Database of single nucleotide polymorphisms dbSNP (*https://www.ncbi.nlm.nih.gov/snp/*), Online Mendelian Inheritance in Man OMIM (*https://www.omim.org/*), Leiden Open variation Database LOVD (*https://www.lovd.nl/*), MITOMAP (*https://www.mitomap.org/MITOMAP*), mtDB (*http://www.mtdb.igp.uu.se/*).

National Library of Medicine (*https://www.nlm.nih.gov)*; Scopus (*https://www.scopus.com)*; EMBASE (*https://www.embase.com/search);* Web of Science (*https://apps.webofknowledge.com)*;) Google Scholar (*https://scholar.google.com)*; Springer (*https://link.springer.com)*; Science Direct (*https://www.sciencedirect.com/)*; gnomAD (*https://gnomad.broadinstitute.or/g)*.

Supplementary data: Methodology: Database Searching, and Inclusion/Exclusion Criteria

The data collection was performed by using "mitochondrial cardiomyopathy/cardiomyopathies" or "mitochondrial diseases with cardiomyopathy/cardiomyopathies" as essential keywords combined with "genetic test/gene testing", "genetic/molecular analysis", "next generation sequencing", "NGS" or "NGS Technology" "Sanger Sequencing", "mtDNA", "mitochondrial DNA" or "nuclear and mitochondrial gene/mutation" as main keywords. Two authors (B.M. and F.B.), independently, have focused literature data based on the titles and abstracts, firstly. Then, full texts were analyzed. Mainly English-language publications (one paper was in French language) were considered. We also searched through the references of the relevant papers, to ensure that no other important articles were missed. Furthermore, we selected only papers, which performed clear molecular investigations and where genetic variations were reported as mutations, excluding polymorphisms or uncertain significant variants. We excluded publications without clinical data or full genetic test information.

To reduce risk of bias, papers had to report and discuss type of study, the reasons for enrolment, an exhaustive clinical and instrumental evaluation, the modality of genetic test. Retrospective study, prospective study, case-control study and case reports were included. Studies that presented incomplete data were not included. Any problem, highlighted by an author, concerning data extraction was discussed collectively and a consensus procedure was adopted to harmonize the extraction process. A final document, showed as Table 3 reporting the selected papers, was elaborate and delivered to the co-authors.
